# Author Correction: Immune phenotyping of diverse syngeneic murine brain tumors identifies immunologically distinct types

**DOI:** 10.1038/s41467-025-60656-x

**Published:** 2025-06-12

**Authors:** Jasneet Kaur Khalsa, Nina Cheng, Joshua Keegan, Ameen Chaudry, Joseph Driver, Wenya Linda Bi, James Lederer, Khalid Shah

**Affiliations:** 1 Center for Stem Cell Therapeutics and Imaging, Brigham and Women’s Hospital, Harvard Medical School, Boston, MA 02115 USA; 2 Department of Neurosurgery, Brigham and Women’s Hospital, Harvard Medical School, Boston, MA 02115 USA; 3 Department of Surgery, Brigham and Women’s Hospital, Harvard Medical School, Boston, MA 02115 USA; 4 Harvard Stem Cell Institute, Harvard University, Cambridge, MA 02138 USA

**Keywords:** Cancer, Oncology

Correction to: *Nature Communications* 10.1038/s41467-020-17704-5, published online 06 August 2020

Authors were notified of an image duplication that exists between the two panels (005 and Mut3 tumors) in the Supplementary Fig. 1D. The authors would like to confirm the images in question were correct in the original re-submission of the manuscript and the error happened during the final proofing stage. The correct image has been identified and the error corrected. This alteration does not change the finding of Supplementary Fig. 1 and the article.

## Supplementary information

Revised Supplementary Information

