# Supplementary material for: Immune phenotyping of diverse syngeneic murine brain tumors identifies immunologically distinct types
Source: Nat Commun. 2020 Aug 6;11:3912. doi: 10.1038/s41467-020-17704-5 (PMC7411074; doi:10.1038/s41467-020-17704-5)
Supplement: Supplementary file 2 — Supplementary information [file 41467_2020_17704_MOESM2_ESM.pdf]

# Supplementary Figure 1

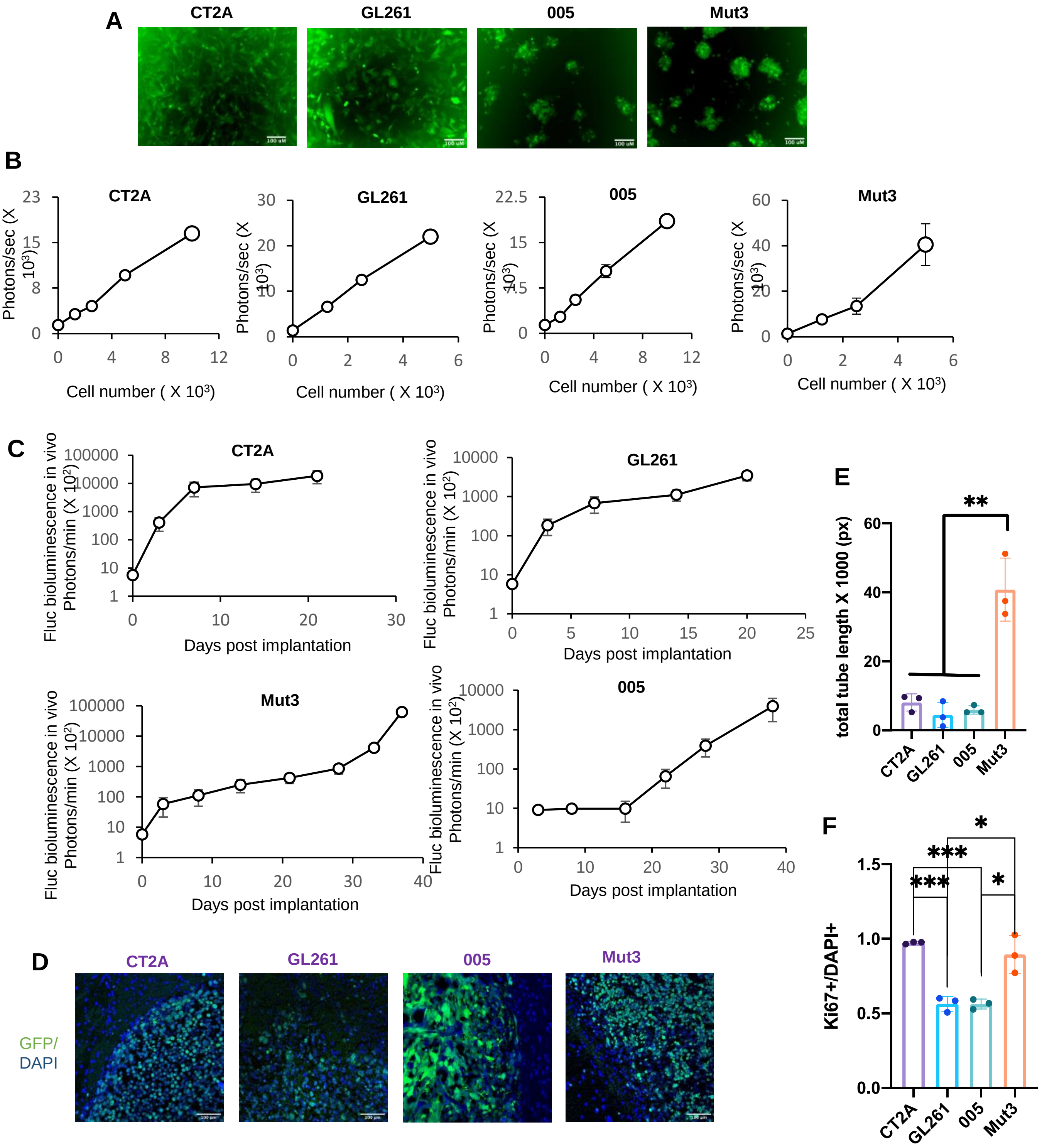


Supplementary Figure 1: A-B:Tumor lines were transduced with lentiviral vector bearing a cDNA fusion of GFP and F-luciferase. Representative images of cells in culture showing GFP expression (A) and firefly luciferase expression detected in vitro in titrating cell number (B). Images are representative of 3 independent experiments and data represented as average ± SE for N=3. Data representative of 3 independent experiments. C: Tumor-growth tracked in vivo by bioluminescence imaging of tumor-bearing mice shows increase in signal over time. Data as average ± SE for N≥ 5. D: GFP expression in brain sections obtained from end-stage tumor-bearing mouse. Images are representative of 3 independent experiments. E: CD31 staining was quantified using wimasis software for tube length. Data represents average ± SE for N ≥ 5.. Two-sided Student's T-test was performed.*p≤ 0.05. F: Ki67+ and DAPI+ cells were counted in 3 random fields in the tumor tissue section and their ratio is reported as average ± SE for 3 images/tumor type. Two-sided Student's T-test was performed.*p≤ 0.05, ***p≤ 0.0005

## Supplementary Figure 2

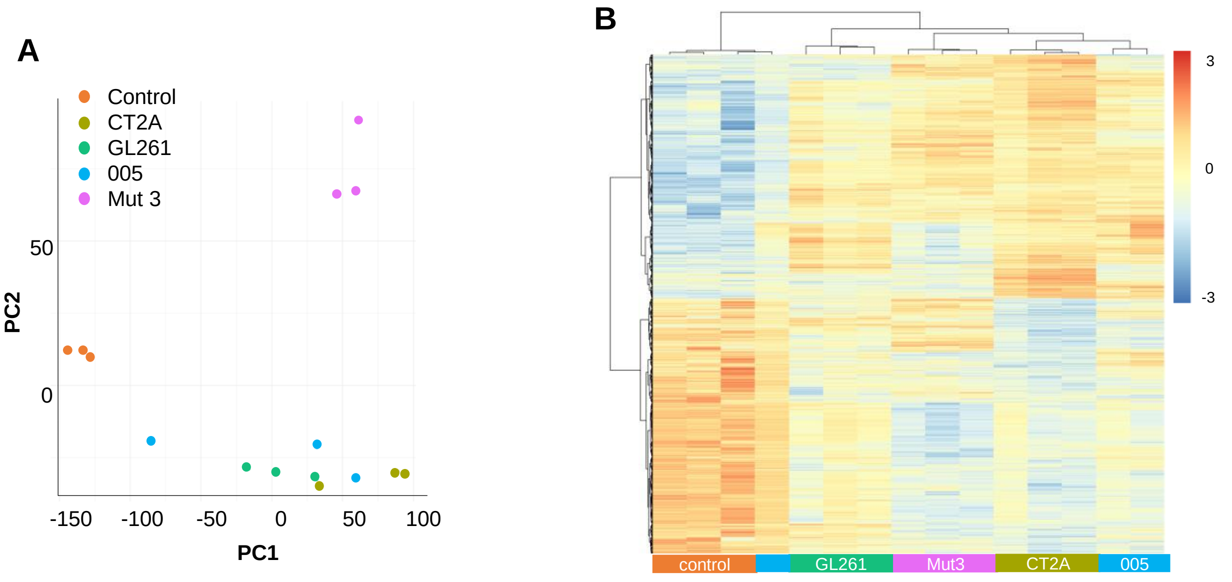


Supplementary Figure 2: RNA sequencing analysis on the RNA isolated from end-stage tumors or control brains of C57bl6 mice (N=3 mice/group). A: Principal component analysis with PC1 and PC2 plotted for control sample and tumor samples. B: Heatmap of top 100 differentially expressed genes.

# Supplementary Figure 3

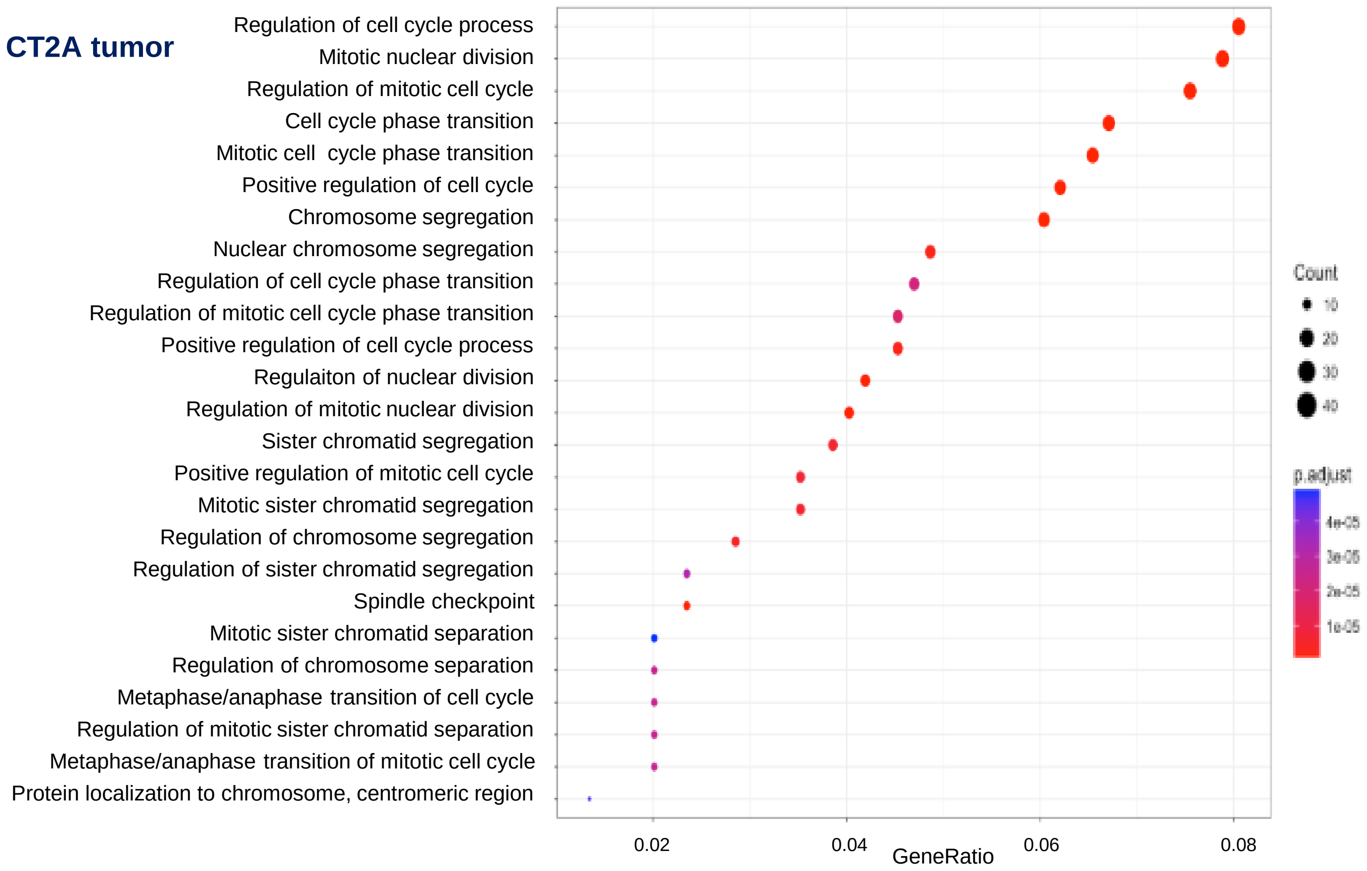


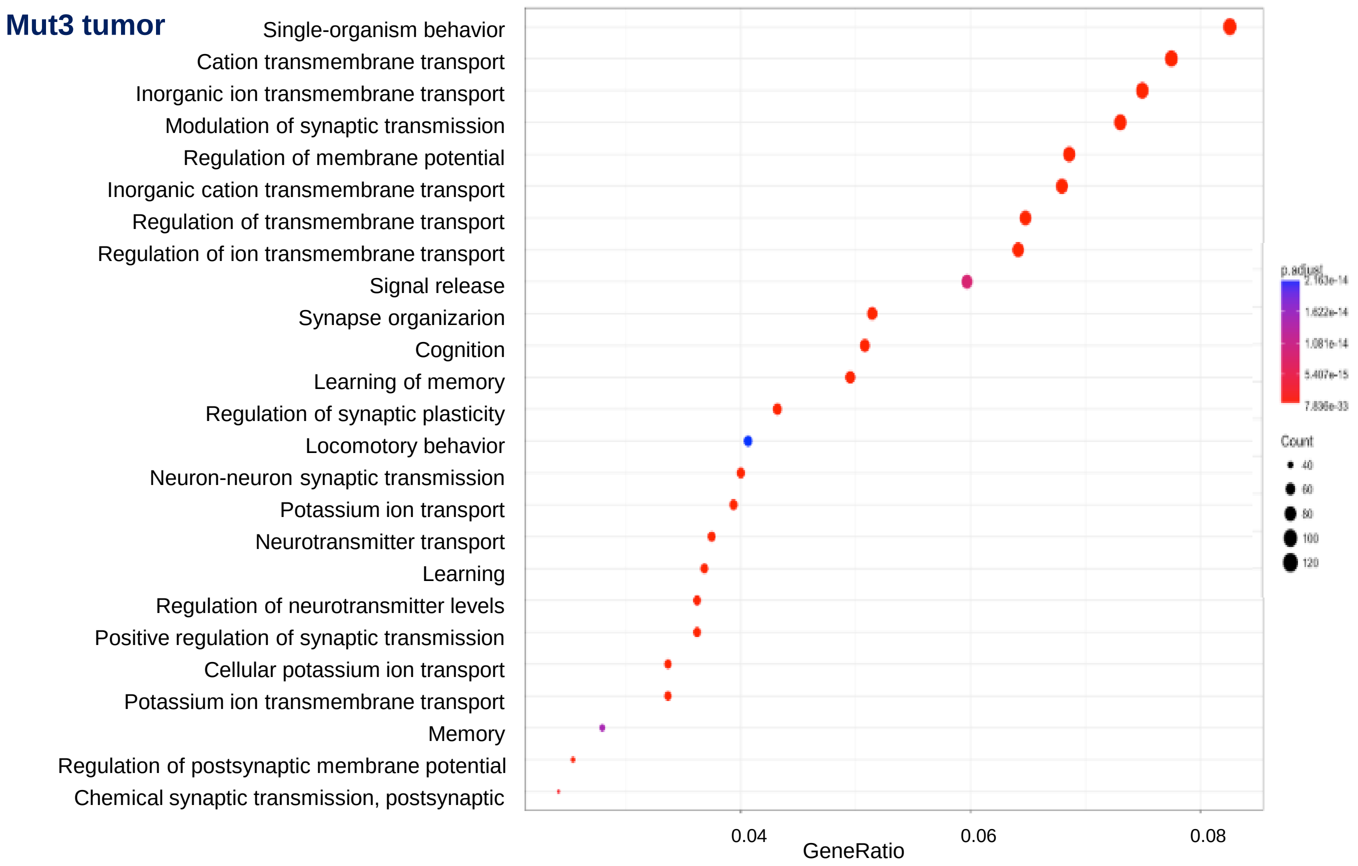


Supplementary Figure 3: Differential expression analysis was performed for each tumor sample using control as a reference. Genes that were significantly different were used for pathway enrichment analysis. Top 25 differentially expressed pathways as compared to naïve brain have been shown for CT2A and Mut3. The statistical test was a hypergeometric test as implemented in the ClusterProfiler R/Bioconductor package (Yu, G. et al, 2012).

# Supplementary Figure 4

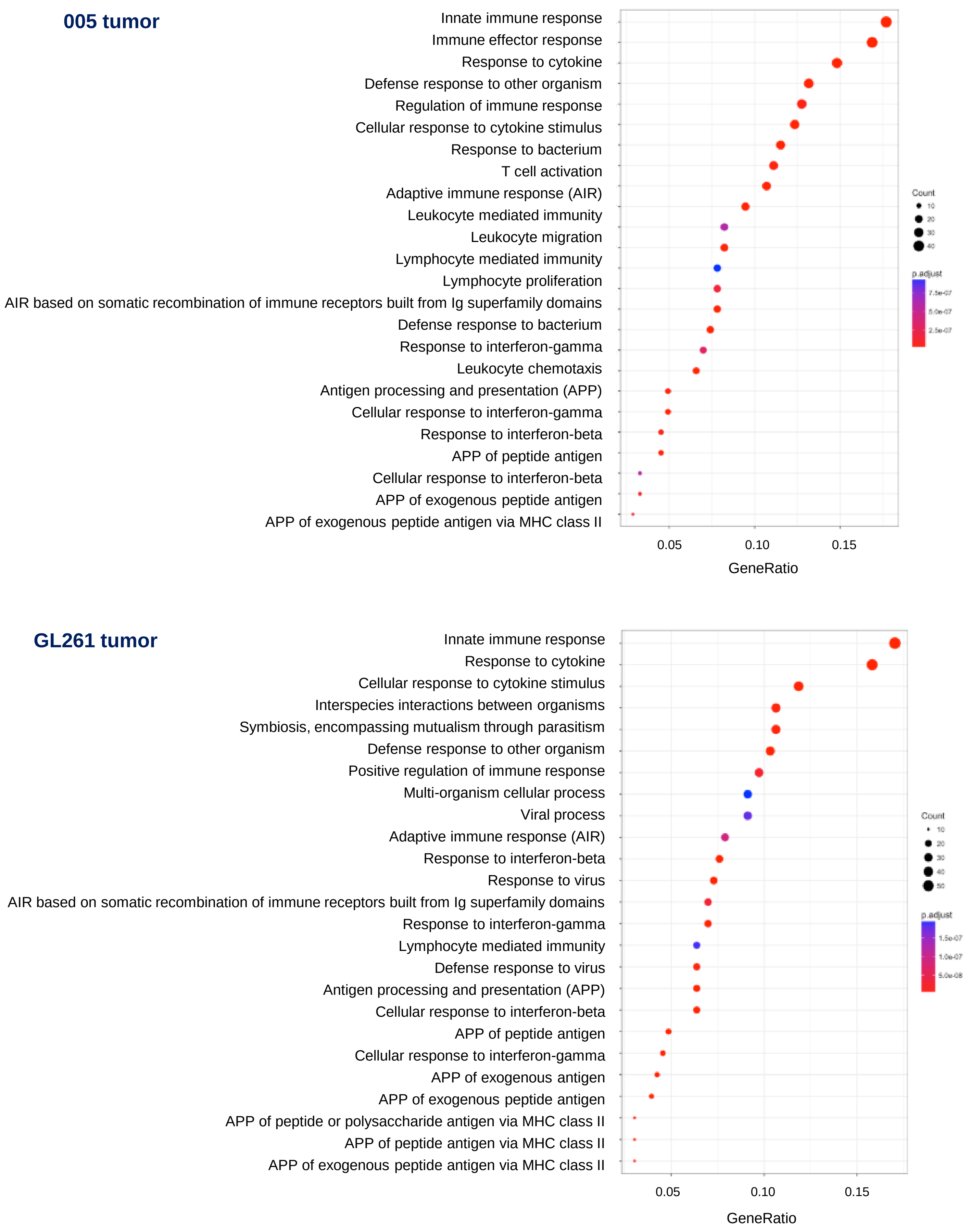


Supplementary Figure 4: Differential expression analysis was performed for each tumor sample using control as a reference. Genes that were significantly different were used for pathway enrichment analysis. Top 25 differentially expressed pathways as compared to naïve brain have been shown for 005 and GL261. The statistical test was a hypergeometric test as implemented in the ClusterProfiler R/Bioconductor package (Yu, G. et al, 2012).

## Supplementary Figure 5

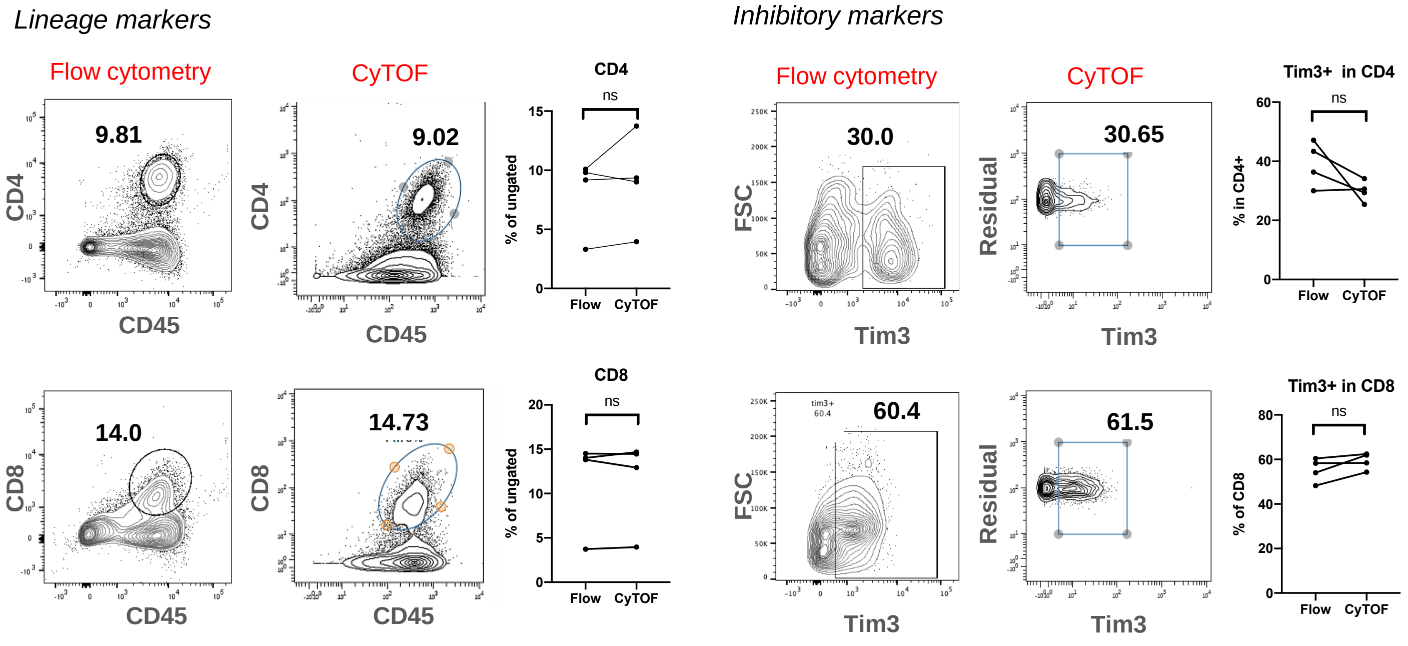


Supplementary Figure 5: Mass cytometry and flow Cytometry comparison. Mixed mouse tumor samples were stained for fluorescent-tagged or metal-tagged antibodies and run through flow cytometer or CyTOF, respectively. Cells were pre-gated for live cells. Gates in each plot show frequency of positive cells. n=4 independent biological replicates. Paired two-sided T-test showed no significant difference. NS= not significant for $p<0.05$.

# Supplementary Figure 6

**A**

| Cell-lineage | | Activation |
|---|---|---|
| CD3 | Ly6G | CD39 |
| CD4 | SiglecF | Lag3 |
| CD8 | CD64 | CTLA-4 |
| CD11b | CD115 | Tim3 |
| CD11c | CX3CR1 | KLRG1 |
| NK1.1 | MHC-II | CD25 |
| CD49b | F4/80 | PD-1H |
| IgM | TCRgd | PD-L1 |
| B220 | CD103 | CD44 |
| Ly6C | | CD62L |
| CD45 | | CD86 |

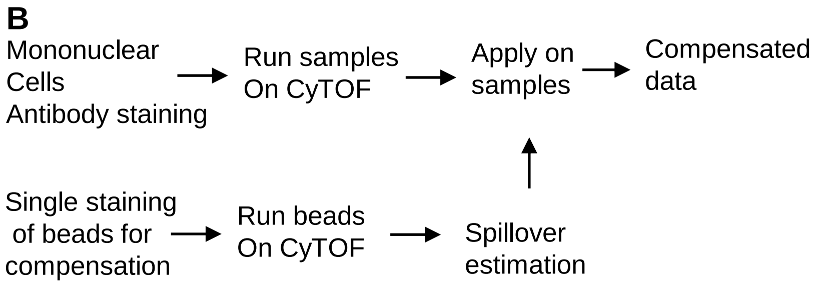


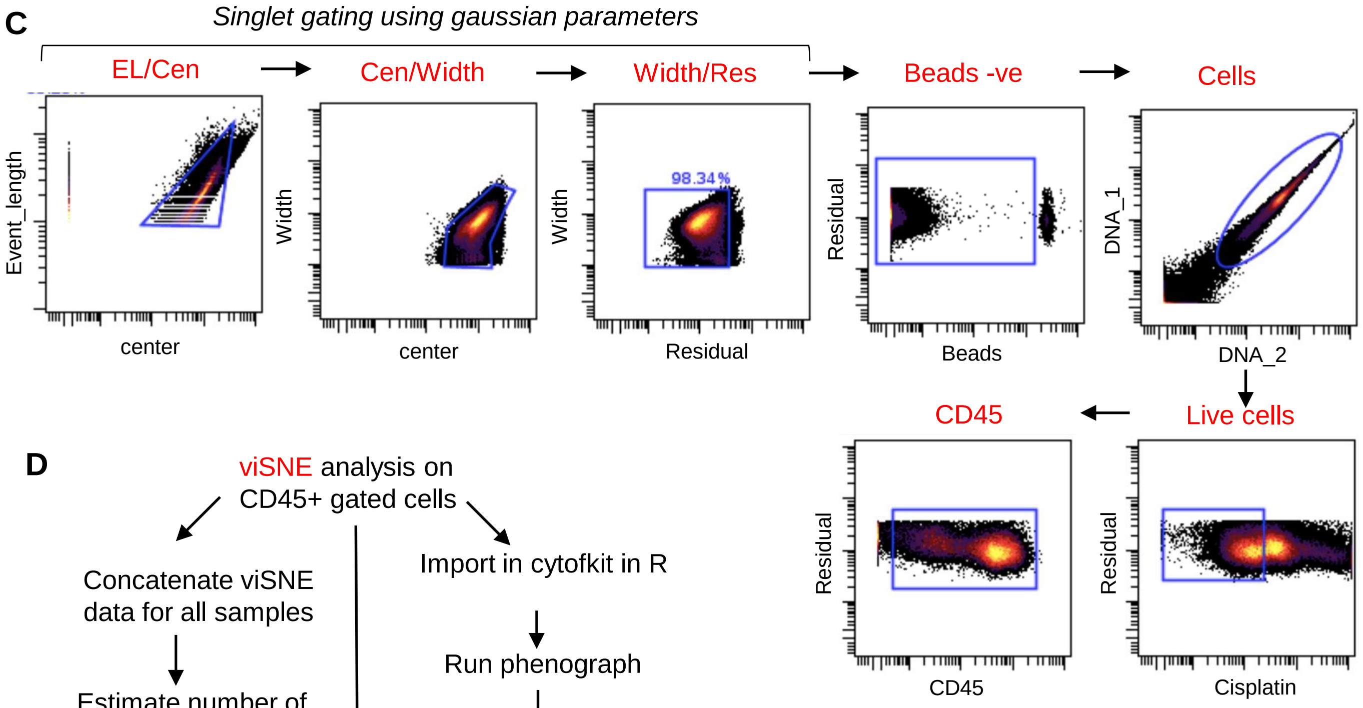


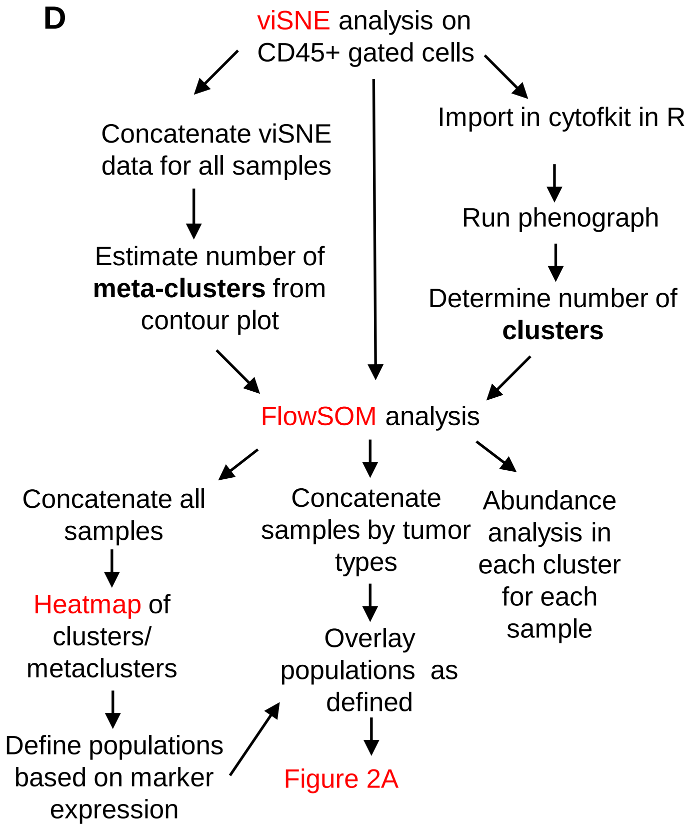


Supplementary Figure 6: A: Antibody panel for anti-murine CyTOF analysis separated into lineage-identifying and activation status markers. B: Schematic representing compensation treatment performed on samples prior to downstream analysis. C: Representative plots for preliminary gating for doublet and dead cell exclusion for samples. D: Schematic demonstrating analysis performed on samples.

## Supplementary Figure 7

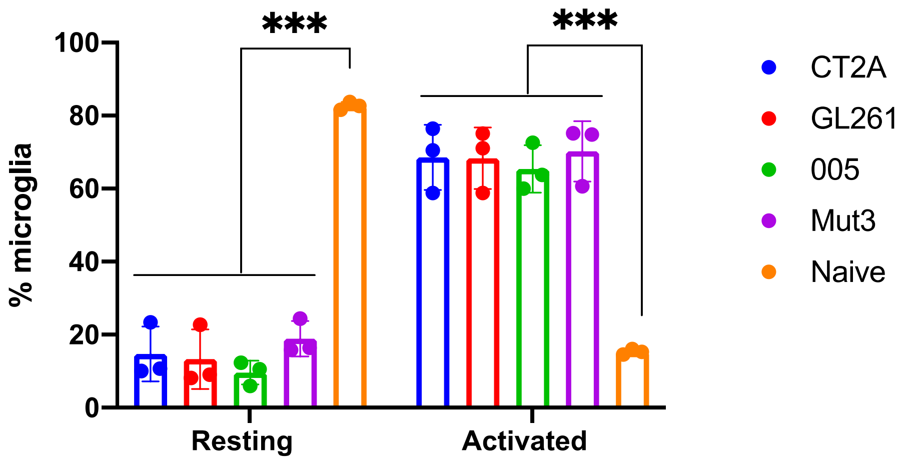


Supplementary Figure 7: Percent population of activated and resting microglia in different tumor types were compared to naïve brain. Data represented as average ± SE for n=3 mice/group. Two-sided student's t-test with Holm-Sidak corrections was applied ***p≤ 0.0005.

# Supplementary Figure 8

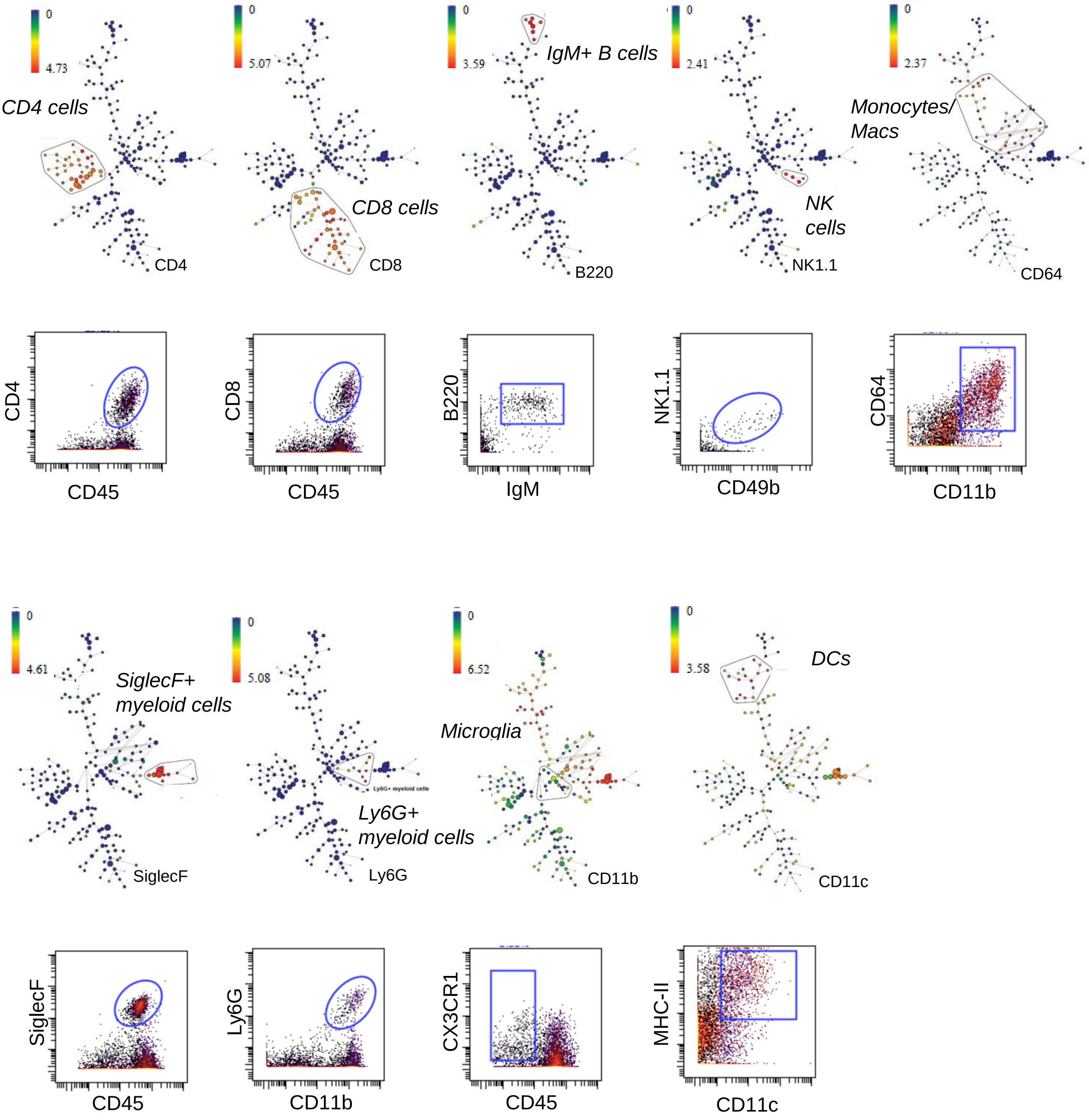


Supplementary Figure 8: A: Markers used for broad population characterization of immune cells in GL261 tumor model were overlaid on a SPADE diagram. Color represents the arc-sinh transformed median expression of each marker. Size of the node represents abundance of population. Flow plots depicting those populations are plotted underneath each SPADE diagram.

## **Supplementary Figure 9**

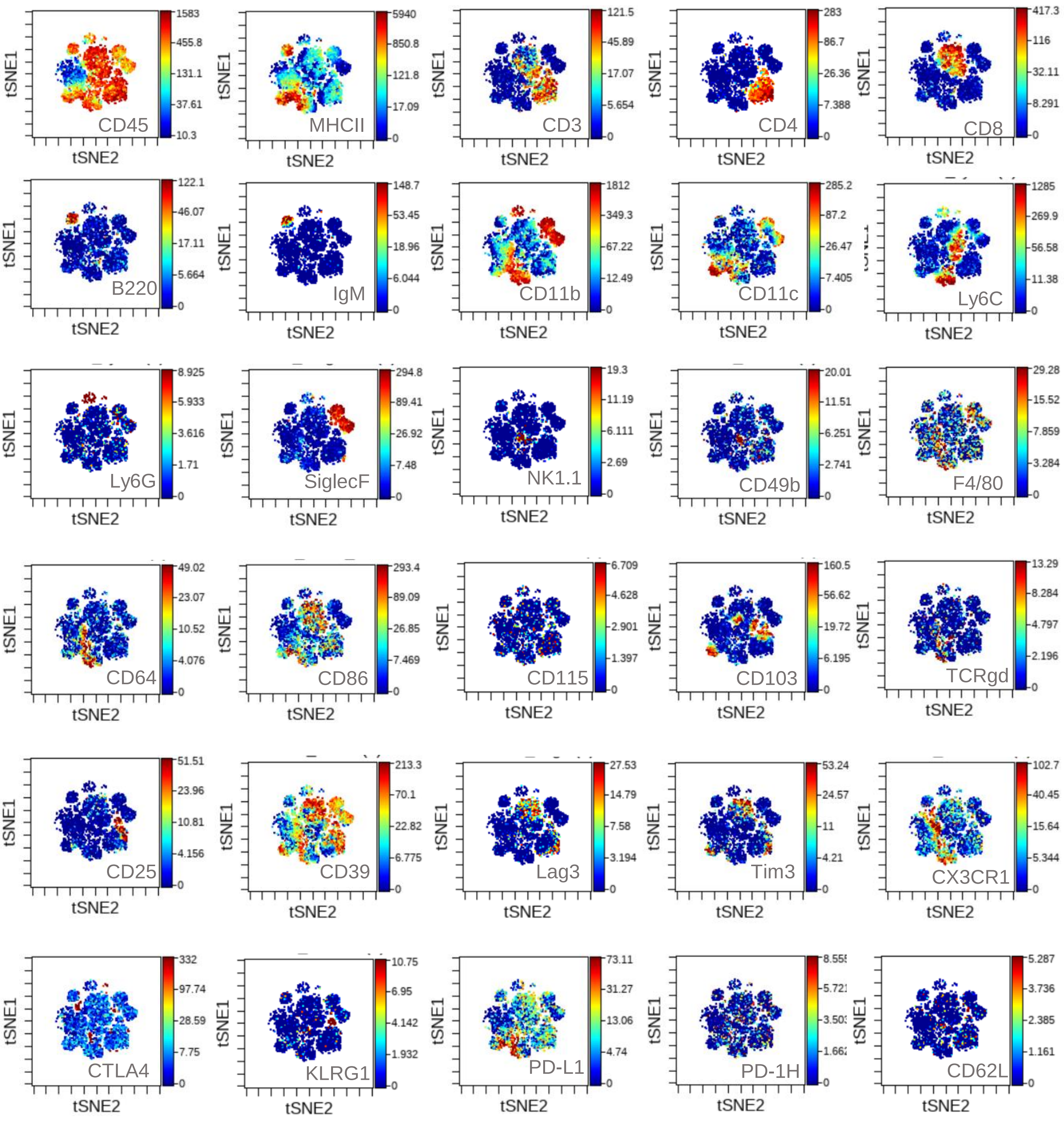


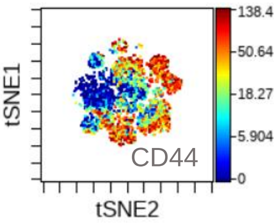


Supplementary Figure 9: Each marker used in CyTOF analysis as listed in Figure S3A overlaid on viSNE plots for GL261 tumor model. Each dot represents a single cell and color gradient correlates with expression level of the defined marker.

## Supplementary Figure 10

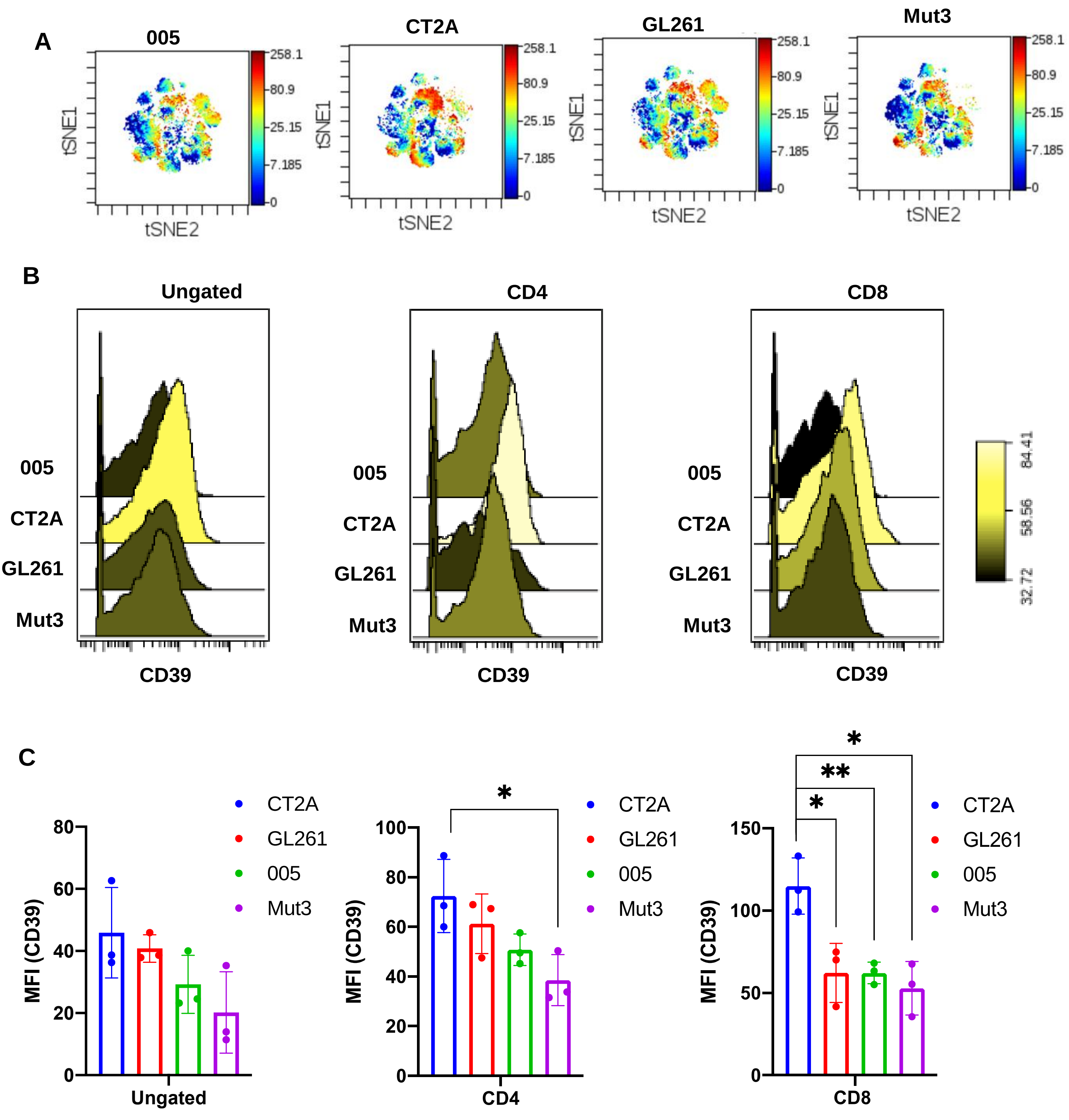


Supplementary Figure 10: A: CD39 expression overlaid on viSNE of concatenated samples of individual tumor types. Each dot represents a single cell and color gradient correlates with expression level of CD39. B: Histogram overlays of CD39 expression in total ungated sample, CD4 and CD8 subsets for the four tumor types. C: Data represented in (B) is plotted as average ± SE for n=3 mice/group. Two-sided Student's t-test was applied. *p≤ 0.05 ,**p≤ 0.005.

# Supplementary Figure 11

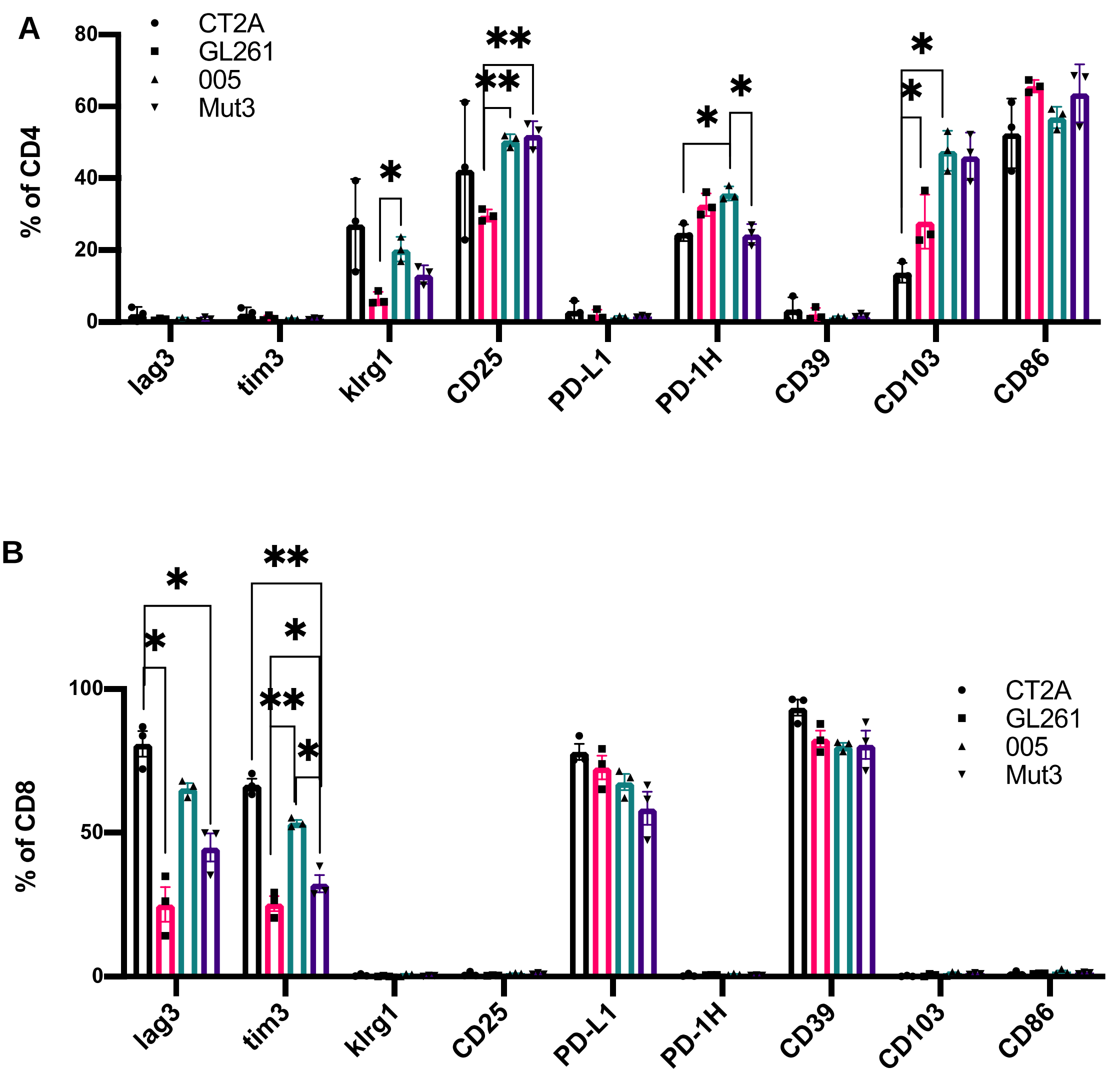


Supplementary Figure 11: Relative percentage of cell populations expressing various T cell function markers in CD4 (A) and CD8 (B) T cell populations in 4 tumor types represented as average ± SE for n=3 mice/group. Two-sided Student's t-test with Holm-Sidak corrections for multiple comparisons was applied. *p≤ 0.05; **p≤ 0.005.

# Supplementary Figure 12

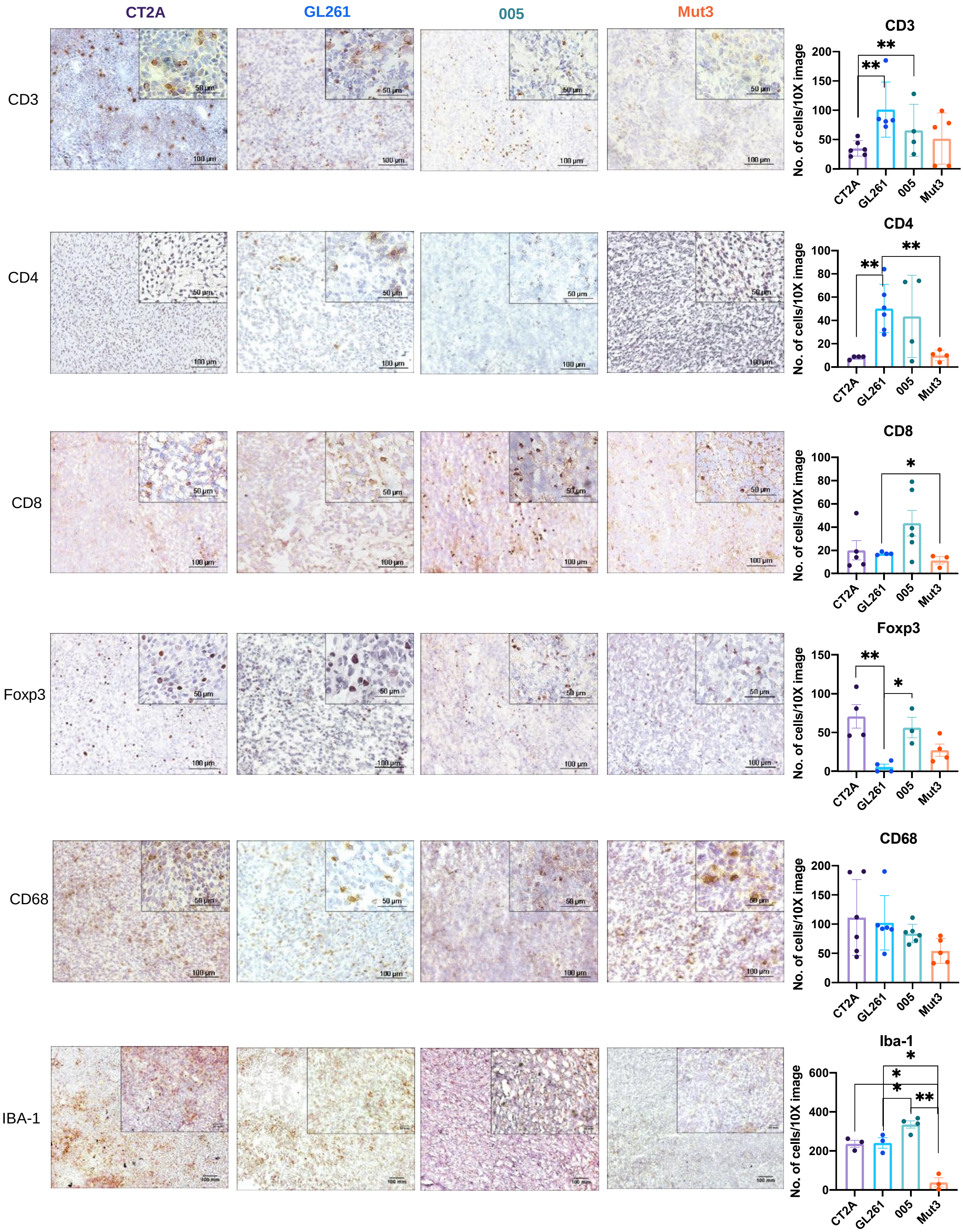


Supplementary Figure 12: Tumors harvested at end stage were sectioned and stained with CD3, CD4, CD8, Foxp3, CD68 and Iba-1. Number of brown cells were counted as positive for each marker in each tumor type and plotted as number of cells identified/field. Data represented as average $\pm$ SE for N $\geq$ 5. Student's t-test with Holm-Sidak corrections for multiple comparisons was applied *p$\leq$ 0.05; **p$\leq$ 0.005.

**Supplementary Figure 13**

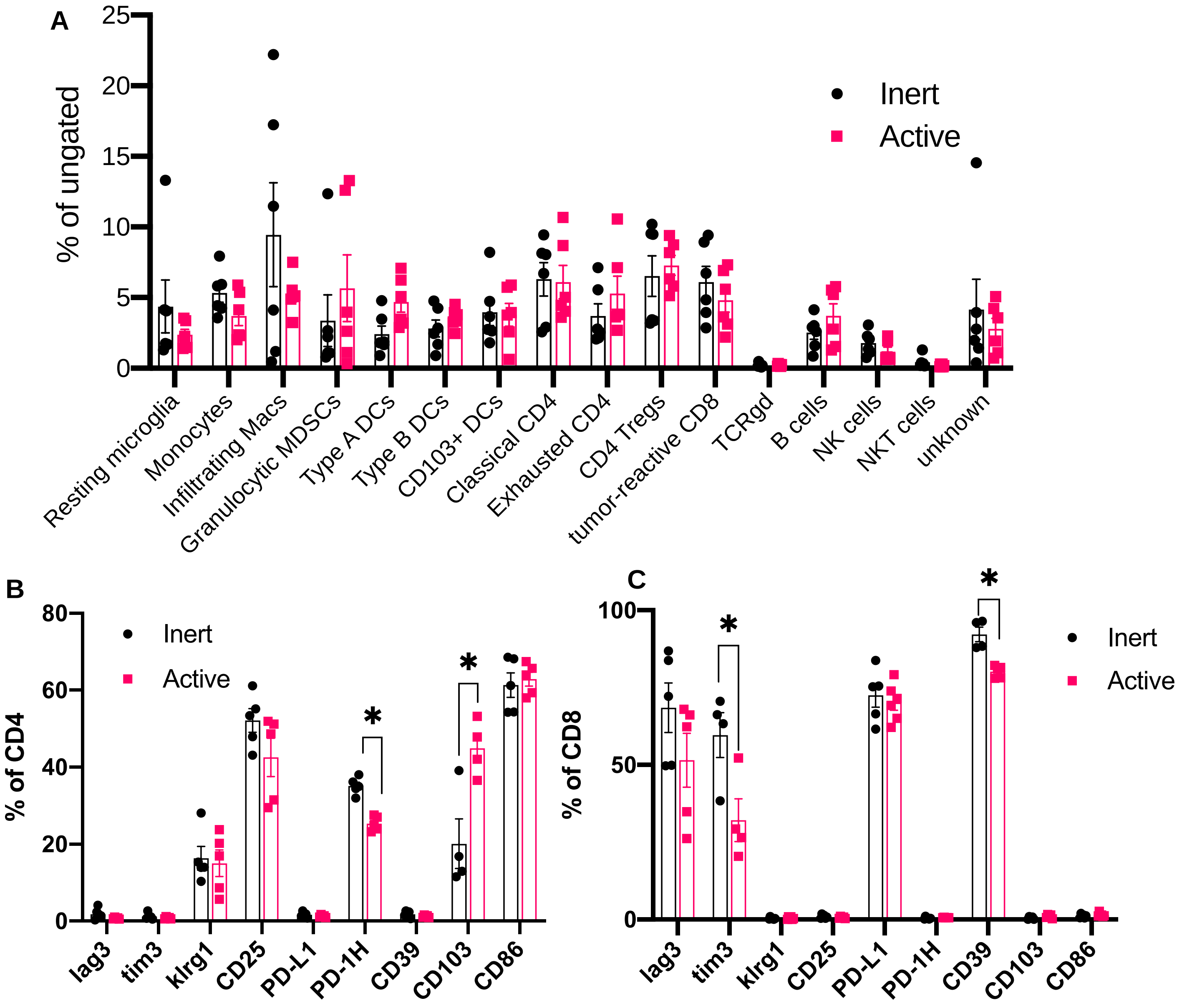


Supplementary Figure 13: Tumors were classified into immunologically active (GL261 and 005) and immunologically silent (CT2A and Mut3) based on RNAseq analysis. FlowSOM analysis was performed followed by abundance analysis. Populations that were not significantly different were plotted. Data is represented as average ± SE for each cluster characterized. B-C: Relative percentage of cell populations expressing various T cell function markers in CD4 (A) and CD8 (B) T cell populations in immunologically inert and active tumor types represented as average ± SE for n=6 mice/group, representative plots from 2 experiments. Two-sided Student's t-test with Holm-Sidak corrections for multiple comparisons was applied *p≤ 0.05.

# Supplementary Figure 14

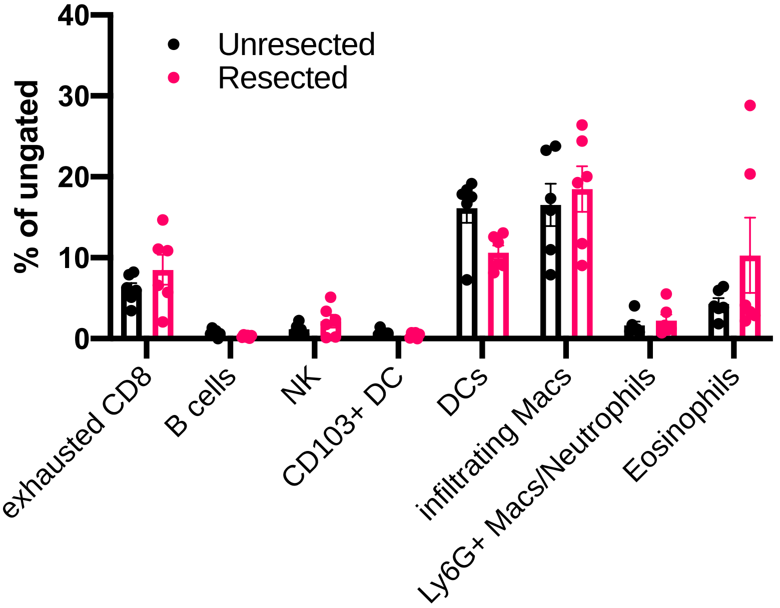


Supplementary Figure 14: Percentage of cells for populations not showing significant differences were plotted as average ± SE (n=6 mice/group, representative plot from 2 independent experiments).

## Supplementary Figure 15

| Cell-lineage | | Activation |
|---|---|---|
| CD3 | CX3CR1 | CD44 |
| CD4 | CD14 | CD279/PD1 |
| CD8 | CD16 | CD39 |
| CD11b | CD115 | CD69 |
| CD11c | CD103 | Tim3 |
| NKp30 | CD86 | CTLA4 |
| CD19 | CD15 | CD25 |
| CD20 | CCR2 | PD-L1 |
| IgM | TMEM119 | CD66b |
| CD172ab | Siglec8 | |
| CD45 | CD68 | |
| CD64 | HLA-DR | |
| CD127 | CD180 | |
| CD33 | CD163 | |
| CD169 | CD304 | |
| Ki67 | STING | |
| Basophil | CCR3 | |

Supplementary Figure 15: Antibody panel for anti-human CyTOF analysis separated into lineage-identifying and activation status markers.

## Supplementary Figure 16

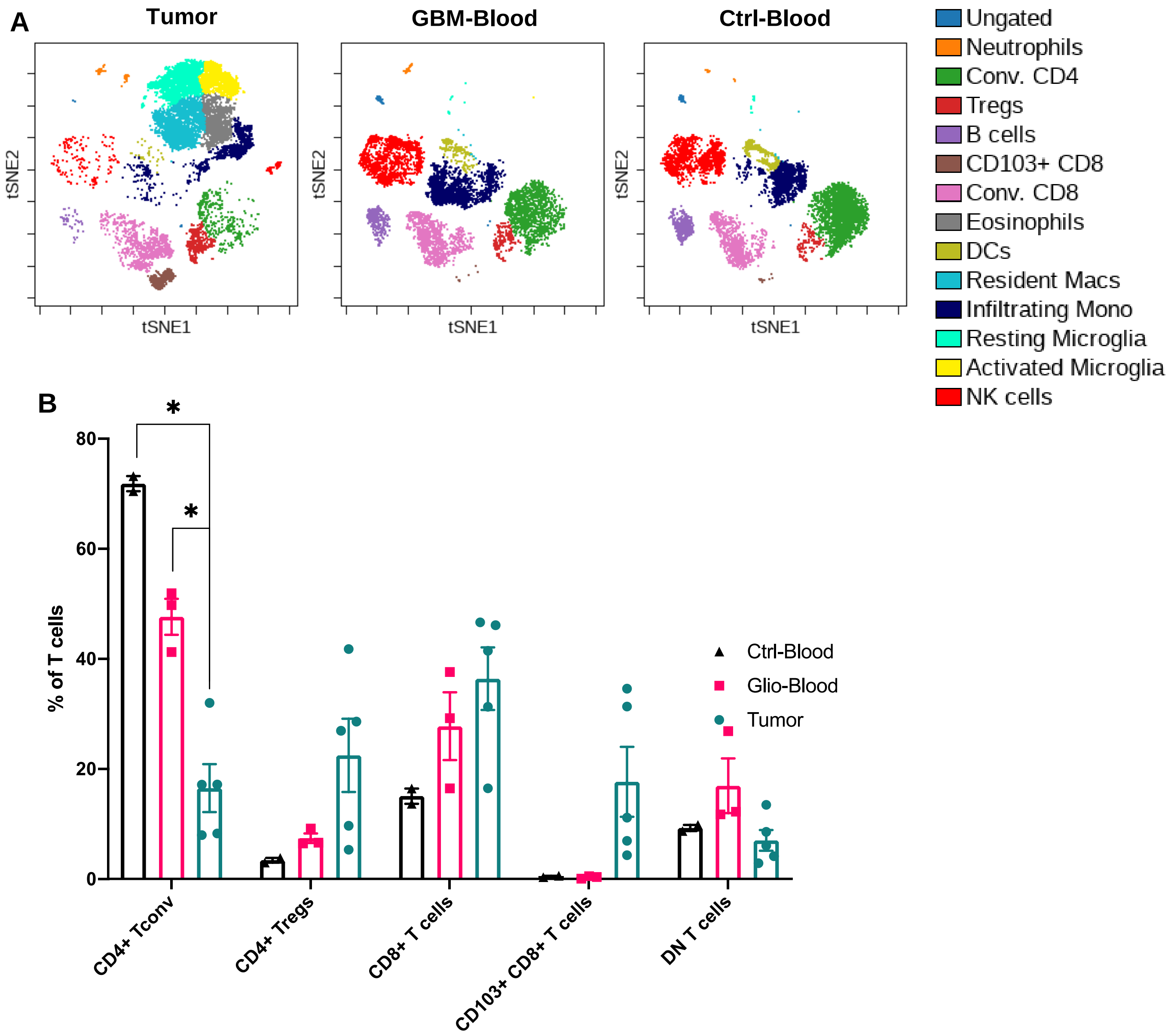


Supplementary Figure 16: CyTOF on isolated lymphocytes from tumor tissue and matched blood from 5 GBM patients and 5 healthy donors was performed. A: FlowSOM analysis for the three tissue types. B: Frequency of T cell subsets were calculated from total T cells GBM tumor tissue, blood from GBM patients and control blood. Data represented as average ± SE for n=5 independent biological repeats. Two-sided Student's t-test with Holm-Sidak corrections for multiple comparisons was applied. *p≤ 0.05.

# Supplementary Figure 17

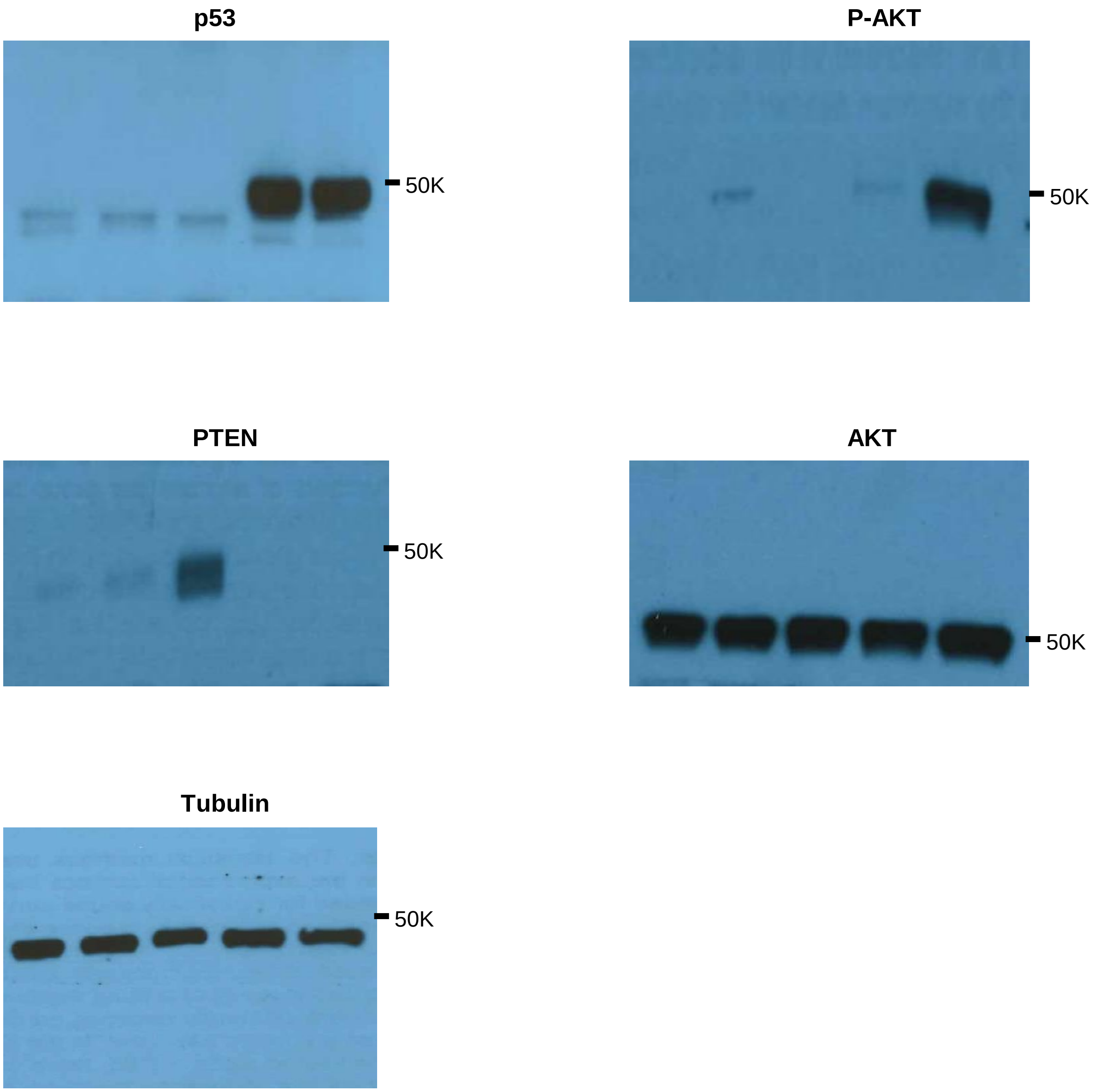


Supplementary Figure 17: Uncropped western blot images shown in Figure 1b
