# Supplementary material for: Immune phenotyping of diverse syngeneic murine brain tumors identifies immunologically distinct types
Source: Nat Commun. 2020 Aug 6;11:3912. doi: 10.1038/s41467-020-17704-5 (PMC7411074; doi:10.1038/s41467-020-17704-5)
Supplement: Supplementary file 3 — Reporting Summary [file 41467_2020_17704_MOESM3_ESM.pdf]

nature research

Corresponding author(s): Khalid Shah

Last updated by author(s): Jun 23, 2020

Reporting Summary

Nature Research wishes to improve the reproducibility of the work that we publish. This form provides structure for consistency and transparency in reporting. For further information on Nature Research policies, see Authors & Referees and the Editorial Policy Checklist .

Statistics

For all statistical analyses, confirm that the following items are present in the figure legend, table legend, main text, or Methods section.

n/a	Confirmed	
☐	☒	The exact sample size (*n*) for each experimental group/condition, given as a discrete number and unit of measurement
☐	☒	A statement on whether measurements were taken from distinct samples or whether the same sample was measured repeatedly
☐	☒	The statistical test(s) used AND whether they are one- or two-sided<br>*Only common tests should be described solely by name; describe more complex techniques in the Methods section.*
☐	☒	A description of all covariates tested
☐	☒	A description of any assumptions or corrections, such as tests of normality and adjustment for multiple comparisons
☐	☒	A full description of the statistical parameters including central tendency (e.g. means) or other basic estimates (e.g. regression coefficient) AND variation (e.g. standard deviation) or associated estimates of uncertainty (e.g. confidence intervals)
☐	☒	For null hypothesis testing, the test statistic (e.g. *F*, *t*, *r*) with confidence intervals, effect sizes, degrees of freedom and *P* value noted<br>*Give P values as exact values whenever suitable.*
☒	☐	For Bayesian analysis, information on the choice of priors and Markov chain Monte Carlo settings
☒	☐	For hierarchical and complex designs, identification of the appropriate level for tests and full reporting of outcomes
☐	☒	Estimates of effect sizes (e.g. Cohen's *d*, Pearson's *r*), indicating how they were calculated

Our web collection on statistics for biologists contains articles on many of the points above.

Software and code

Policy information about availability of computer code

Data collection	No software was used
Data analysis	GraphPad Prism 8.4.0

For manuscripts utilizing custom algorithms or software that are central to the research but not yet described in published literature, software must be made available to editors/reviewers. We strongly encourage code deposition in a community repository (e.g. GitHub). See the Nature Research guidelines for submitting code & software for further information.

Data

Policy information about availability of data

All manuscripts must include a data availability statement. This statement should provide the following information, where applicable:

- Accession codes, unique identifiers, or web links for publicly available datasets
- A list of figures that have associated raw data
- A description of any restrictions on data availability

The datasets generated during the current study are available from the corresponding author on reasonable request.

Field-specific reporting

Please select the one below that is the best fit for your research. If you are not sure, read the appropriate sections before making your selection.

☒ Life sciences ☐ Behavioural & social sciences ☐ Ecological, evolutionary & environmental sciences

For a reference copy of the document with all sections, see nature.com/documents/nr-reporting-summary-flat.pdf

Life sciences study design

All studies must disclose on these points even when the disclosure is negative.

Sample size	Our study involved immune-phenotyping of mouse tumors and GBM patients by mass cytometry. These experiments are difficult to perform, laborious and expensive. We got meaningful data that was reproducible as independent experiments with a N=3-5.
Data exclusions	No data was excluded from the analysis
Replication	All attempts at replication were successful.
Randomization	Tumor-bearing mice were randomized for the resection experiment where half were resected and the other half were left un-resected.
Blinding	Immuno-histochemistry slides were coded and blinded for the marker and tumor type for the person counting number of positive cells.

Reporting for specific materials, systems and methods

We require information from authors about some types of materials, experimental systems and methods used in many studies. Here, indicate whether each material, system or method listed is relevant to your study. If you are not sure if a list item applies to your research, read the appropriate section before selecting a response.

Materials & experimental systems

n/a	Involved in the study
☐	☒ Antibodies
☐	☒ Eukaryotic cell lines
☒	☐ Palaeontology
☐	☒ Animals and other organisms
☐	☒ Human research participants
☒	☐ Clinical data

Methods

n/a	Involved in the study
☒	☐ ChIP-seq
☐	☒ Flow cytometry
☒	☐ MRI-based neuroimaging

Antibodies

Antibodies used	Tim3-PE (clone-RMT3-23; biolegend; Catalog number-119703); CD4-BV711 (clone-RM4-5, biolegend, Catalog number-100549), CD8-PerCP (clone-53-6.7, biolegend, catalog number-100731)
Validation	All antibodies were titrated to determine suitable concentration. flow cytometry plots for these antibodies are present in the manuscript.

Eukaryotic cell lines

Policy information about cell lines

Cell line source(s)	005 tumor cells were obtained from Dr. Hiroaki Wakimoto, MGH, Boston. Mut3 cells were received from Dr. Sean Lawler, BWH, Boston. GL261 and CT2A cells were present in the lab.
Authentication	Cell lines were genotyped and western blot data is present in Fig 1B.
Mycoplasma contamination	All cell lines used were negative for mycoplasma
Commonly misidentified lines (See ICLAC register)	none of the listed misidentified cell lines has been used.

Animals and other organisms

Policy information about studies involving animals; ARRIVE guidelines recommended for reporting animal research

Laboratory animals	C57/Bl6 mice, 6-8 week old, females
Wild animals	study did not involve wild animals
Field-collected samples	study did not include samples collected from fields.
Ethics oversight	BWH animal care committee

Note that full information on the approval of the study protocol must also be provided in the manuscript.

Human research participants

Policy information about studies involving human research participants

Population characteristics	Human Research participants were diagnosed with WHO grade-IV GBM. Tumor tissue isolated post-resection were genotyped and were identified to be IDH WT.
Recruitment	The brain tumor samples were collected under 10-417, an institutional banking IRB approved protocol. The samples were distributed to our lab under tissue subusage protocol approval. All patients undergoing a brain tumor surgery at the Brigham are open to this banking protocol at the time of surgery.
Ethics oversight	DF/HCC IRB

Note that full information on the approval of the study protocol must also be provided in the manuscript.

Flow Cytometry

Plots

Confirm that:

- [x] The axis labels state the marker and fluorochrome used (e.g. CD4-FITC).
- [x] The axis scales are clearly visible. Include numbers along axes only for bottom left plot of group (a 'group' is an analysis of identical markers).
- [x] All plots are contour plots with outliers or pseudocolor plots.
- [x] A numerical value for number of cells or percentage (with statistics) is provided.

Methodology

Sample preparation	Brain was minced with a scalpel on ice in calcium supplemented 1X HBSS (GIBCO) supplemented with 1mg/ml Collagenase IV (Sigma) and 0.25 mg/ml DNase I (Sigma) and incubated for 1 hour with intermittent shaking at 37°C. Tumor Infiltrating immune cells (TIICs) were separated by 30% and 70% percoll gradient centrifugation. Isolated TTICs were stained with live/dead fixable violet dye followed by antibody staining. Cells were fixed with 4% paraformaldehyde and stored until running them on the flow cytometer.
Instrument	BD Fortessa
Software	FlowJo
Cell population abundance	The manuscript does not have any sorting experiments.
Gating strategy	A generous FSC/SSC gate was applied followed by negative gate for live/dead violet.

- [x] Tick this box to confirm that a figure exemplifying the gating strategy is provided in the Supplementary Information.
